# TNFR2 promotes pancreatic cancer proliferation, migration, and invasion via the NF-κB signaling pathway

**DOI:** 10.18632/aging.204941

**Published:** 2023-08-16

**Authors:** Zetian Gao, Qiubo Zhang, Hang Chen, Jiayi Chen, Jingyu Kang, Hang Yu, Yufei Song, Xie Zhang

**Affiliations:** 1 The Affiliated Lihuili Hospital, Ningbo University, Ningbo, Zhejiang 315040, China; 2 Ningbo Clinical Pathology Diagnosis Center, Ningbo, Zhejiang 315211, China; 3 Health Science Center, Ningbo University, Ningbo, Zhejiang 315211, China

**Keywords:** pancreatic ductal adenocarcinoma, TNFR2, NF-κB, survival analysis

## Abstract

Purpose: Pancreatic ductal adenocarcinoma (PDAC) is a highly aggressive malignant disease with low overall survival; chemotherapy and immunotherapy have limited efficacy. Tumor necrosis factor receptor 2 (TNFR2), a type II transmembrane protein, contributes to the development and progression of several tumors. In this study, we elucidated the effect and molecular mechanisms of TNFR2.

Method: We used The Cancer Genome Atlas and the Genotype-Tissue Expression database to compare the expression of the TNFR2 gene between normal and malignant pancreatic tissue. Using immunohistochemical staining, we divided the patients into high and low-expression groups, then investigated clinicopathologic data and survival curves of pancreatic cancer patients. We measured TNFR2 protein expression in PANC-1 and ASPC-1 pancreatic cancer cells subjected to TNFR2 small interfering RNA or negative control treatment. We performed proliferation, invasion, and migration assays to study the biological effects of TNFR2 in PDAC. The molecular mechanisms were validated using western blotting.

Results: TNFR2 was more highly expressed in PDAC cells and tissues than controls. Abundant expression of TNFR2 was associated with aggressive clinicopathologic characteristics and poor outcomes. Overexpression of TNFR2 promoted PDAC cell proliferation, migration, and invasion *in vitro*. Mechanistically, TNFR2 binds to TNF-α and activates the NF-κB signaling pathway.

Conclusion: TNFR2 is a prognostic marker that facilitates the proliferation, migration, and invasion of PDAC via the NF-κB signaling pathway. TNFR2 may become a therapeutic target.

## INTRODUCTION

Pancreatic ductal adenocarcinoma (PDAC) is highly aggressive. PDAC has a low early detection rate and a 5-year overall survival rate of less than 8% [1]. Access to health care was adversely affected by coronavirus disease 2019, which led to a decreased incidence but increased mortality [2]. PDAC patients are usually asymptomatic in the early stage, which delays early diagnosis and effective treatment. Surgery is the only curative therapy; however, fewer than 10% of patients undergo surgical resection, and most patients are diagnosed in a metastatic or locally-advanced stage. Chemotherapy is the primary treatment for advanced PDAC, and the standard first-line chemotherapy regimen is albumin paclitaxel, gemcitabine, and folfirinox [3]; however, the curative effects were unsatisfactory. In recent years, immunotherapy has shown promising results in preclinical studies for PDAC treatment; nevertheless, outcomes have not been substantially improved, primarily due to the complex tumor microenvironment [4].

Tumor necrosis factor-α (TNF-α) is a cytokine that mediates pro-inflammatory responses and regulates immunity. TNF-α exerts biological effects by combining tumor necrosis factor receptor 1 (TNFR1) and tumor necrosis factor receptor 2 (TNFR2), a type II transmembrane protein in the tumor necrosis factor receptor superfamily [5]. TNFR2 is expressed in T lymphocytes, myeloid suppressor cells, endothelial cells, cardiomyocytes, oligodendrocytes, and thymocytes, where it participates in anti-inflammatory processes and immune regulation [6].

Researchers previously focused on the relationship between TNFR2 and regulatory T cells (Tregs) [7]. TNFR2 is highly expressed on Tregs in the tumor microenvironment (TME). TNFR^+^Treg exerts a master function on Treg cell activation and expansion in the tumor microenvironment and the primary cellular mechanism that assists tumor immune escape [8]. Targeting several cell surface proteins, including TNFR2, can enhance the antitumor immune response by eliminating Treg cells. Further studies showed that in TNFR2 knockout mouse models of colon (MC-38) and lung (H-59) cancer, liver metastases from tumors were significantly reduced, as well as the number of intrahepatic myeloid-derived suppressor cells (MDSCs) and Treg cells was significantly reduced [9]. Chopra et al. using a luciferase-expressing homozygous B16-F10 mouse model of melanoma lung metastasis, verified that exogenous TNF induced the expansion of Treg cells via TNFR2 and promoted melanoma lung metastasis. In contrast, lung metastasis and intrapulmonary Treg cells were significantly reduced in mice lacking TNF or TNFR2 on immune cells [10]. Recent studies have revealed that TNFR2 is not only expressed on the surface of Treg cells to promote inflammation, immune regulation, and tumor metastasis but also expressed on tumor cells as a cancer-promoting protein and enhances the proliferation and metastasis of tumor cells [11].

NF-κB is an inducible transcription factor discovered 30 years ago. Since then, it has been found to perform several roles in the genetic induction of cellular responses. NF-κB regulates critical biological processes such as immune responses, inflammation, cell adhesion, survival, cell cycle, and apoptosis. Previous studies found that immune cells can secret tumorigenic cytokines to increase NF-κB abundance in inflammatory cells/tissues and ultimately contribute to tumorigenesis [12]. Previous research indicated that TNFR2 facilitated the infiltration and function of Tregs through the NF-κB pathway [13].

Currently, there are no studies elucidating the role and functional mechanism of TNFR2 in pancreatic cancer. This study evaluated TNFR2 expression in human pancreatic cancer tissues and cells. Furthermore, we analyzed the relationship between TNFR2 expression and clinicopathological parameters and explored the effect and mechanism of TNFR2 in pancreatic cancer cells.

## METHODS

### Patients and specimens

Seventy human pancreatic cancer tissues and ten normal tissues were obtained from patients who underwent surgical resection of the pancreas at the Affiliated Lihuili Hospital between 1 July 2017 and 1 July 2022. The enrollment criteria were: primary diagnosis of pancreatic ductal adenocarcinoma; no chronic infections; no rheumatoid autoimmune diseases; no severe cardiac, hepatic, or renal dysfunction. Affiliated Lihuili Hospital, Ningbo University’s clinical research ethics committee approved the study.

### Cell culture

Normal pancreatic duct epithelial cells hERT-HPNE and pancreatic cancer cell PANC-1 were purchased from BNCC (China). Pancreatic cancer cells (ASPC-1) were purchased from Cox9x (China). The hTERT-HPNE and ASPC-1 cells were cultured in Roswell Park Memorial Institute-1640 with 10% fetal bovine serum (FBS, Bovgen, Australia). The PANC-1 cells were cultured in high-glucose Dulbecco’s modified Eagle’s medium with 10% FBS.

### Gene expression profiling interactive analysis (GEPIA)

GEPIA (http://gepia.cancer-pku.cn/) is a publicly accessible database that contains gene expression of PDAC patients downloaded from The Cancer Genome Atlas (TCGA) and the Genotype-Tissue Expression (GTEx) database. These databases total include 179 pancreatic tumor tissues and 171 normal pancreatic tissues.

### Cell transfection

Small interfering RNA (siRNA) target sequences were verified on the human TNFR2 sequence. TNFR2 siRNA and negative control (NC) siRNA sequences were designed and produced by GenePharma (Shanghai, China). TNFR2 siRNA sequences were designed as follows: 5′-GGUCCUUCAAGUUAGCUCATT-3′. Cell transfections were performed using Lipofectamine 2000 kit (Invitrogen, Carlsbad, CA, USA). Lipofectamine 2000 and siRNA were added to Opti-MEM™ (Gibco, Grand Island, NY, USA) and mixed for 20 mins. Then, the mixture was added to PDAC cells cultured in basal medium. After six hours, the basal medium was replaced with complete medium. The effect of transfection was determined by western blot after three days.

### Quantitative reverse transcription-polymerase chain reaction (qRT-PCR)

The PCR primers were as follows: TNFR2, forward: 5′-ACACGCAGCCAACTCCAGAA-3′, and reverse: 5′-TGATGACACAGTTCACCACTCCTAT-3′; GAPDH, forward: 5′-GCACCGTCAAGGCTGAGAAC-3′, and reverse: 5′-TGGTGAAGACGCCAGTGGA-3′. Total RNA was extracted from hERT-HPNE, PANC-1, and ASPC-1 cells using a TransZol Up Plus RNA Kit (Transgen, China). Total RNA was reverse transcribed to cDNA using a TransScript All-in-One First-Strand cDNA Synthesis SuperMix for qPCR kit (Transgen, China). The qRT-PCR was performed using PerfectStart Green qPCR SuperMix Kit (Transgen, China) in ABI 7500 Fast Real-Time PCR system (Applied Biosystems, Foster City, CA, USA) to measure TNFR2 mRNA expression.

### Western blot

RIPA cell lysis buffer (Solarboi, China), containing 1% PMSF and 1% phosphatase inhibitor cocktail (Cwbio, China), was used to cleave proteins for 15 mins on ice. The lysed cells were centrifuged at 12000 rpm for 15 min, and the supernatants were collected for protein quantification using a BCA Protein Assay kit (Beyotime, China). The total protein was separated by 7.5% SDS-PAGE (NCM Biotech, China) and transferred to 0.45-μm PVDF membranes (Millipore, Burlington, MA, USA). The membranes were washed with blocking solution (Yoche, Shanghai, China) for 10 mins and incubated on a shaker at 4°C overnight with diluted TNFR2 (1:1000, ab109322, Abcam, UK), β-Tubulin, (1:1000, A12289, ABclonal, China), phospho-IKK alpha/beta (1:1000, AF3013, affinity), phospho-IκBα (1:1000, AP0707, ABclonal, China), NF-κB (1:1000, A19653, ABclonal, China), phospho-NF-κB(1:1000, AP0124, ABclonal, China). Membranes were washed in 0.1% Tris-buffered saline Tween-20 and incubated in goat anti-rabbit IgG H&L (HRP) (1:2,000, LF102; Epizyme, China) for two hours. Finally, protein bands were visualized using WesternBright ECL (Apgbio, China) and quantified using Image J.

### IHC

Pancreatic tissue was fixed with 4% paraformaldehyde, dehydrated, and embedded in paraffin. Embedded tissue sections were rehydrated using stepped alcohol washes. The sections were incubated with primary antibody against TNFR2 (1:250, ab109322, Abcam) followed by secondary antibody (Yongnian, China). Two pathologists rated IHC scores between 0 and 3 blindly and independently. The IHC scores were: no staining (−), weak staining (+), moderate staining (++), and intense staining (+++).

### Cell proliferation assay

We seeded 2000 PDAC cells/well in 96-well plates for 24 hours. Cells were transiently transfected with siRNA. We added 10 μl Cell Counting Kit-8 solution (Transgen, China) to each well at 0, 1, 2, 3, and 4 days after transfection. The 96-well plates were cultured at 37°C in 5% CO_2_ for 2 hours. Absorbance was determined using a microplate reader set at a 450 nm wavelength. Cell proliferation was calculated using absorbance.

### Wound-healing assay

PDAC cells were seeded in six-well plates and transiently transfected with siRNA. When the cell density was 100%, a 200-μl pipette tip created a scratch on the monolayer. After the cells were washed in phosphate-buffered saline three times, the complete medium was replaced with a serum-free medium. Scratch photos were recorded at 12 hours using an IX71 inverted microscope (Olympus Corporation) and analyzed using ImageJ and GraphPad Prism 5 software.

### Transwell cell migration and invasion assays

PDAC cells were transiently transfected with siRNA and were seeded in 24-well Transwell chambers (8-μm pore size; Corning, NY, USA) to determine the migratory and invasive capacities. For migration assays, 5 × 10^4^ PDAC cells were placed in 200 μl of serum-free medium in the upper chamber, and 700 μl of medium containing 20% FBS was added to the lower chamber. For the invasion assays, chambers were inserted with 50 μl of 1:8 mixture of Matrigel (ABW, Shanghai, China) and basal medium for three hours in a 37°C incubator. Then, 5 × 10^4^ PDAC cells were seeded in 200 μl of serum-free medium in the upper chamber, and 700 μl of medium containing 20% FBS was added to the lower chamber. After the cells were incubated for 48 h, 4% paraformaldehyde was used to fix the cells for 20 mins, and crystal violet was used to stain the fixed cells for 15 min. The migratory and invasive photos were recorded in five random 100× microscopic fields using an IX71 inverted microscope. ImageJ was used to count the stained cells in photographs of five 100× microscopic fields, and the average number of cells was calculated.

### Statistical analysis

Statistical analyses were performed using GraphPad Prism 5 software and SPSS 26. *In vitro* experimental data were analyzed using the Student’s *t*-test. Clinicopathological data were analyzed using the chi-square test. Kaplan-Meier survival analysis was performed for TNFR2. *P*-values less than 0.05 were considered statistically significant; *p*  <  0.05 was designated by ^*^, *p*  < 0.01 by ^**^, and *p* < 0.001 by ^***^.

### Data availability

The data supporting this study’s findings are available on request from the corresponding author.

## RESULTS

### TNFR2 is overexpressed in PDAC cell lines and tissues

**Figure 1:**
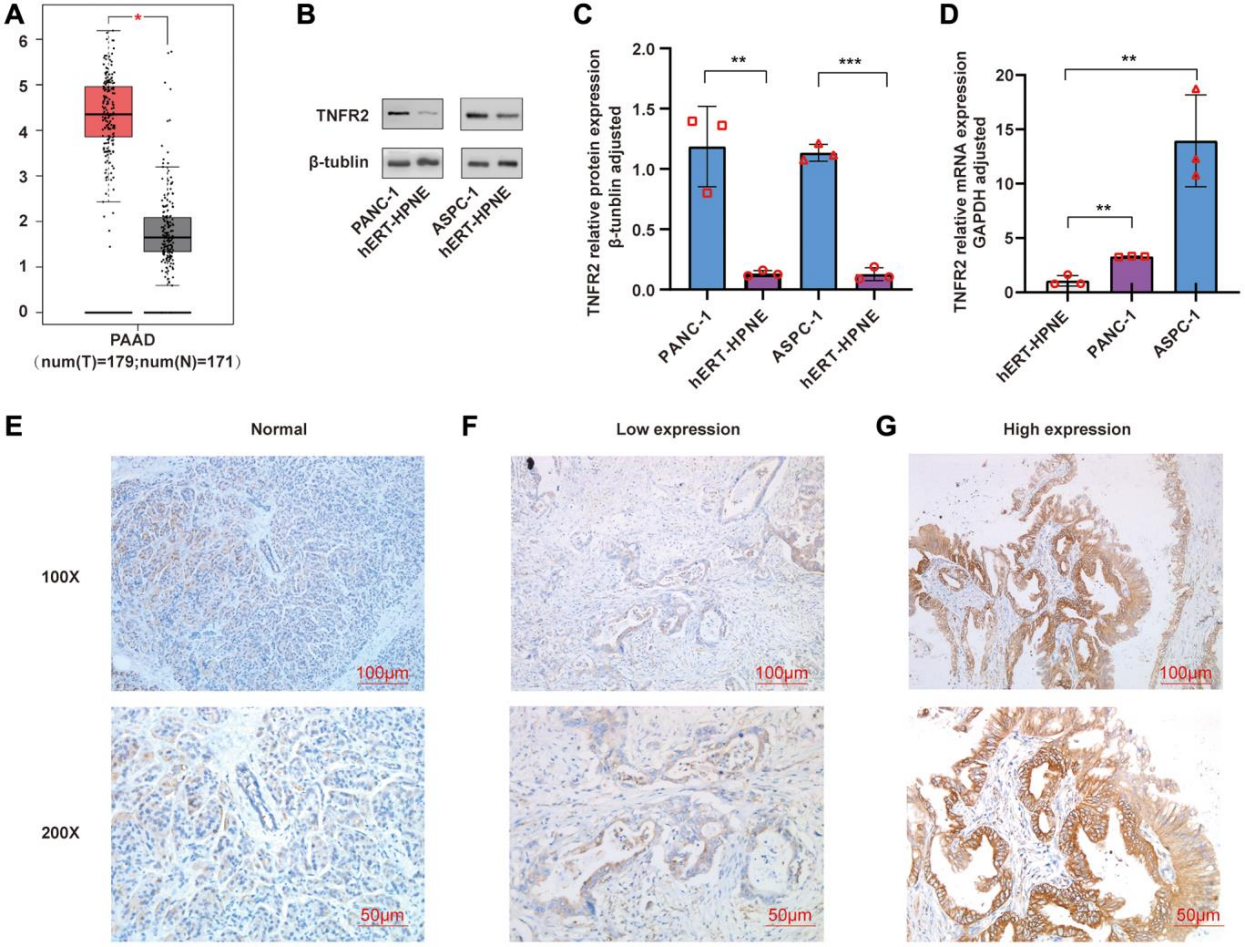
(**A**) TNFR2 gene was analyzed using GEPIA. (**B**, **C**) TNFR2 protein in PANC-1 and ASPC-1 cells were measured by western blot. (**D**) TNFR2 mRNA in PANC-1 and ASPC-1 cells were detected using qRT-PCR. (**E**) Immunohistochemical staining in normal pancreatic tissue. (**F**) Immunohistochemical staining in the low-expression group of pancreatic cancer tissue. (**G**) Immunohistochemical staining in the high-expression group of pancreatic cancer tissue.

TNFR2 gene in TCGA and GTEx were analyzed using GEPIA. As shown in Figure 1A, the TNFR2 gene was overexpressed in pancreatic cancer tissues (*p* < 0.05). We measured the TNFR2 expression in PDAC cell lines using western blot and qRT-PCR. Compared with the normal pancreatic duct epithelial cell hERT-HPNE, protein (Figure 1B, 1C) and mRNA (Figure 1D) expression were significantly higher in pancreatic cancer cell line PANC-1 and ASPC-1.

Expression of TNFR2 was evaluated in 70 pancreatic cancer tissues and 10 normal tissues using immunohistochemical staining. We mainly observed TNFR2 staining in pancreatic ducts. TNFR2 was barely expressed in normal pancreatic tissue (Figure 1E); however, TNFR2 was expressed in the ducts of pancreatic cancer tissue to varying levels (Figure 1F, 1G). We clustered groups according to the IHC score: low-expression group (−, +, ++) and high-expression group (+++). As shown in Table 1, the expression of TNFR2 in pancreatic cancer tissues was significantly higher than in the normal pancreatic tissues (*p* < 0.05).

**Table 1:** Expression of TNFR2 in normal subjects and PDAC patients.

	**Low**	**High**	***p***
Pancreatic cancer tissue	33	37	0.028^*^
Pancreatic normal tissue	9	1	

### TNFR2 expression is associated with PDAC proliferation, metastasis, and survival

TNFR2 was expressed in the ducts of pancreatic cancer tissue to varying levels and we divided 70 PDAC patients into two groups by TNFR2 IHC score: low-expression group (+, ++) and high-expression group (+++). In 70 pancreatic cancer specimens, the low-expression group accounted for 47.1%, and the high-expression group accounted for 52.9% (Table 1). Clinicopathological data of PDAC patients were collected, including sex, location, age, tumor size, lymph node metastasis, distant metastasis, and pathologic grade.

**Figure 2:**
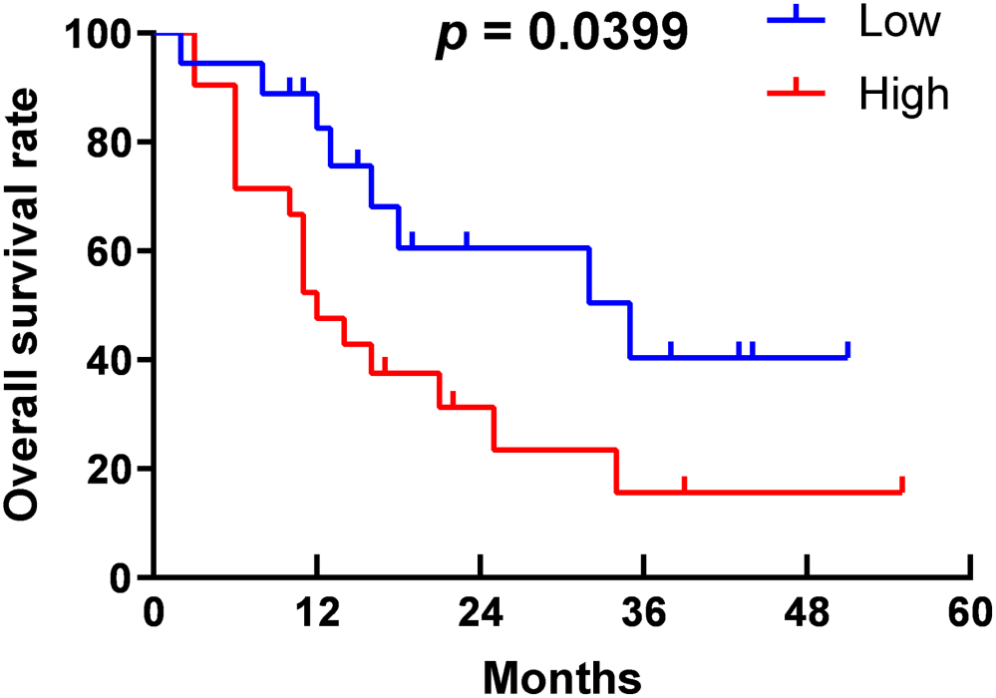
Survival curve of PDAC patients.

As shown in Table 2, in the high-expression group, 23 of 37 specimens (62.2%) had large tumor size, which was more than 12 of 33 specimens (36.4%) in the low-expression group, and the difference was statistically significant (*p* = 0.031). Sixteen patients (43.2%) in the high-expression group had lymph node metastasis, which was much more than five patients (15.2%) in the low-expression group (*p* = 0.01); 23 of 37 cases (62.2%) in the high-expression group had a low differentiation degree, more than eight of 33 cases (24.2%) in the low-expression group (*p* = 0.001). These findings suggest that high-expression of TNFR2 is positively related to large tumor size, lymph node metastasis, and low differentiation degree. Otherwise, the results indicated no significant difference in age, gender, location, or distant metastasis between the groups. PDAC patients are usually asymptomatic in the early stage, which delays early diagnosis, resulting in metastasis and loss of the opportunity to undergo surgery. Therefore, only nine patients had distant metastasis in our cohort, and the small sample size may influence the statistical difference between the two groups. Furthermore, we followed pancreatic cancer patients who underwent surgery. Survival was displayed in Kaplan-Meier curves (Figure 2). Patients in the high-expression group had poorer outcomes than those in the low-expression group (*p* = 0.0399). This finding indicated that high-expression of TNFR2 is positively associated with poor outcomes in PDAC. Overall, we demonstrate that high-expression of TNFR2 is related to aggressive clinicopathologic characteristics and poorer prognosis.

**Table 2:** Clinicopathological characteristics of normal subjects and PDAC patients.

	**Low**	**High**	***p***
Age (years)			
<70	18 (54.5)	22 (59.5)	0.678
≥70	15 (45.5)	15 (40.5)	
Location			
Head, neck	12 (41.4)	21 (72.7)	0.08
Body, tail	17 (58.6)	12 (27.3)	
Sex			
Male	25 (75.8)	27 (73.0)	0.79
Female	8 (24.2)	10 (27.0)	
Tumor size			
≤3 cm	21 (63.6)	14 (37.8)	0.031^*^
>3 cm	12 (36.4)	23 (62.2)	
Lymph node metastasis			
No	28 (84.8)	21 (56.8)	0.01^*^
Yes	5 (15.2)	16 (43.2)	
Distant metastasis			
No	30 (90.9)	31 (83.8)	0.595
Yes	3 (9.1)	6 (16.2)	
Pathologic grade			
Poor	8 (24.2)	23 (62.2)	0.001^*^
Intermediate + high	25 (75.8)	14 (37.8)	

### TNFR2 downregulation suppresses pancreatic cancer cell proliferation

**Figure 3:**
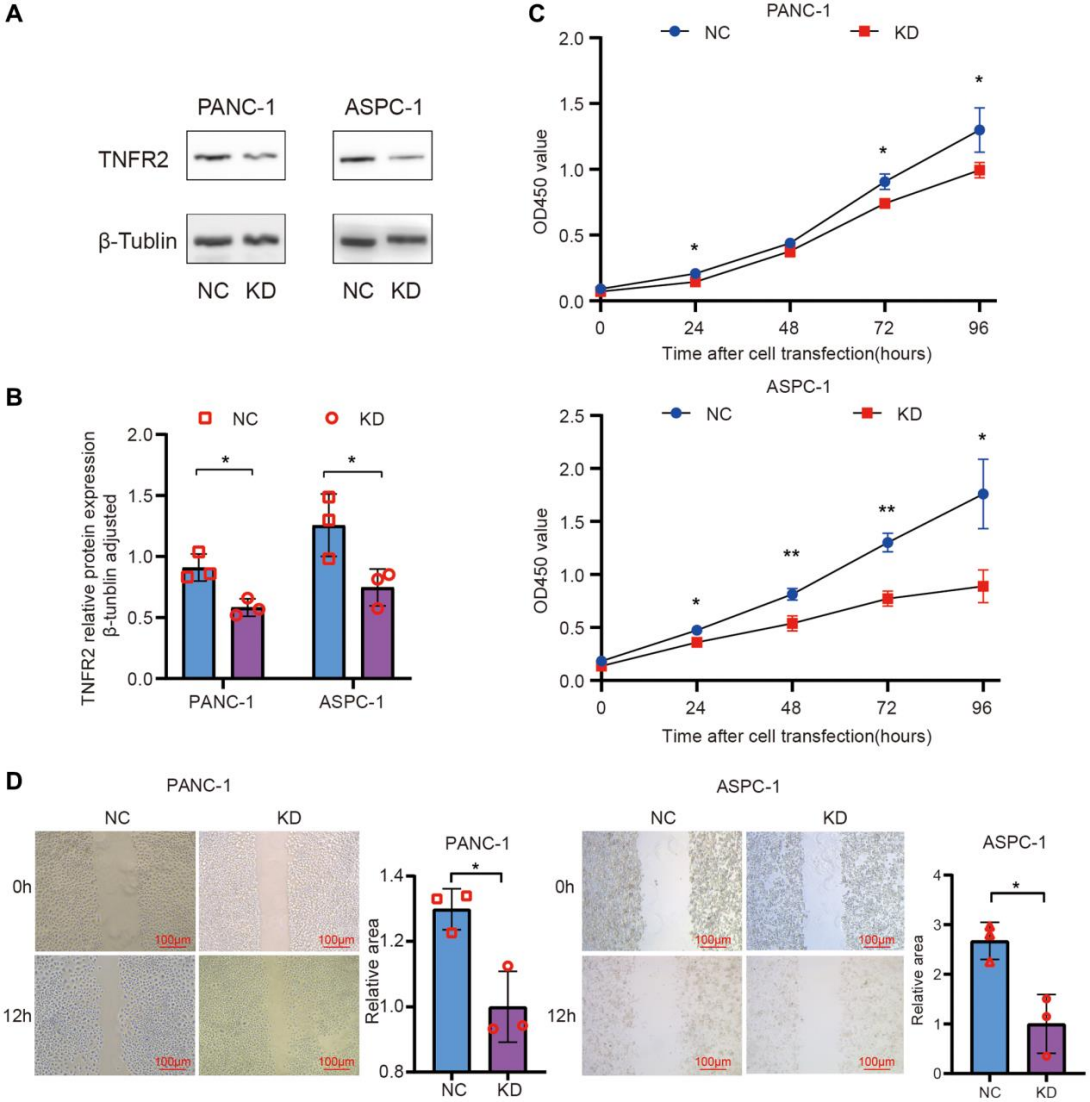
**Knockdown of TNFR2 in the PANC-1 and ASPC-1 cells.** (**A**, **B**) TNFR2 protein in the PANC-1 and ASPC-1 cells after transfection with NC-siRNA and TNFR2 siRNA sequences. (**C**) PANC-1 and ASPC-1 cell viability were determined using the CCK-8 assay. (**D**) Wound-healing assays determined migratory ability.

We knocked down TNFR2 in PANC-1 and ASPC-1 cell lines using siRNA transfection. NC siRNA and TNFR2-siRNA sequences were transfected into PANC-1 and ASPC-1 cells. The PDAC cells were divided into negative control (NC) and knockdown (KD) groups. TNFR2 protein levels were determined 72 h after transfection using western blot analysis. As is shown in Figure 3A, 3B, siRNA inhibited TNFR2 protein levels.

To determine whether TNFR2 affects pancreatic cancer cell proliferation, cell viability was measured using the CCK-8 assay at 0, 1, 2, 3, and 4 days after transfection. Compared to the NC group, the viability in the KD group was significantly lower in PANC-1 and ASPC-1 cells (Figure 3C). Especially on days 3 and 4, viability significantly differed between the NC and KD groups (*p* < 0.05). These findings suggest that reducing TNFR2 contributes to the inhibition of PDAC cell proliferation.

### TNFR2 downregulation suppresses pancreatic cancer cell migration and invasion

**Figure 4:**
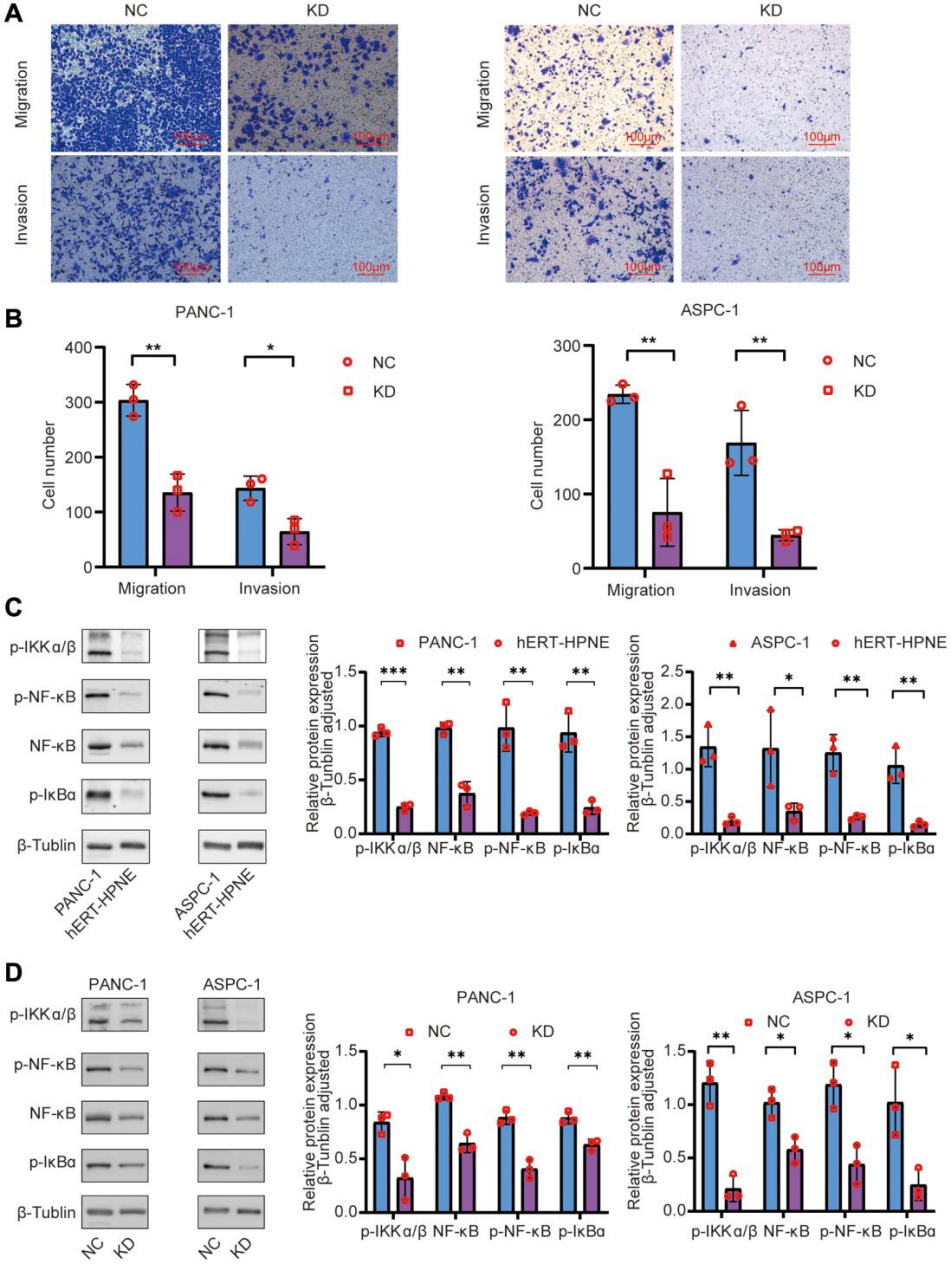
(**A**, **B**) Effects of TNFR2 knockdown on migratory and invasive capacities using Transwell assays in PANC-1 and ASPC-1 cells. (**C**) Western blotting determined that NF-κB signaling-related proteins were overexpressed in the PANC-1 and ASPC-1 cells. (**D**) Western blotting determined that NF-κB signaling-related proteins were downregulated after TNFR silencing in PANC-1 and ASPC-1 cells.

Wound-healing assays were used to measure cell migration changes by calculating the wound closure area (Figure 3D). The KD group’s healing area was smaller than the NC group in PANC-1 and ASPC cells (*P* < 0.05). Transwell assays were used to verify the migration and invasion results to determine the role of TNFR2 in regulating the metastatic procession of pancreatic cancer cells, including migration and invasion. For the migration assay, NC and KD PDAC cells were seeded on the upper layer of the chamber. After 12 h, we randomly selected five visual fields at 100× magnification for counting and statistics. Figure 4A, 4B shows that TNFR2 downregulation reduced migration in PANC-1 and ASPC-1 cells. Matrigel was inserted into chambers for the migration assay to assess the role of TNFR2 downregulation on invasiveness. After 24 h, the number of cells in the lower chamber of the KD group was significantly less than that of the NC group (Figure 4A, 4B). TNFR2 downregulation significantly suppressed invasive capacity in PANC-1 and ASPC-1 cells. These findings suggest that TNFR2 is required for pancreatic cancer cell metastatic progression.

### TNFR2 exerts acts via the NF-kB signaling pathway

TNFR2 affects PDAC cell biology; however, the molecular mechanisms remain unknown. Compared with the normal pancreatic duct epithelial cell hERT-HPNE, western blotting showed protein levels of p-IKKα/β, NF-κB, p-NF-κB, p-IκBα increased in pancreatic cancer cell lines (Figure 4C), suggesting that the NF-κB signaling pathway was upregulated in PDAC cells. To investigate the relationship between TNFR2 and the NF-κB signaling pathways, we silenced TNFR2 and measured p-IKKα/β, NF-κB, p-NF-κB, p-IκBα protein in the NC and KD groups. Western blot analysis showed that these proteins in the KD group decreased compared with those in the NC group (Figure 4D). These results suggest that TNFR2 promotes biological properties in PDAC cells through the NF-κB signaling pathway.

## DISCUSSION

Approximately 80% of pancreatic ductal adenocarcinoma patients are diagnosed in advanced or metastatic stages and lose the opportunity for surgery. Chemotherapy is the preferred treatment for non-surgical patients; however, drug resistance and toxicities limit their efficacies [14]. Therefore, uncovering novel targets and determining molecular mechanisms are critical to improving survival.

TNFR2 gene is more abundant in pancreatic cancer tissues than normal ones. IHC revealed that TNFR2 protein was highly expressed in 33 of 70 paraffin specimens; however, it was barely expressed in normal pancreatic tissues. Western blotting and qRT-PCR revealed that TNFR2 was overexpressed in pancreatic cancer cells, which were compatible with the results of GEPIA and IHC in PDACs. In summary, TNFR2 is overexpressed in pancreatic tumors and may be essential in developing pancreatic cancer.

Recent studies statistically showed elevated expression of TNFR2 in 25 types of tumors, including renal cancer, multiple myeloma, ovarian cancer, colon cancer, and cutaneous T-cell lymphoma [8]. An immunohistochemistry (IHC) study showed that TNFR2 expression in esophageal squamous cell carcinoma tissue was higher than in normal esophageal tissue, and high expression of TNFR2 implies deep invasion, poor differentiation and low overall survival [15]. TNFR2 was overexpressed in human non-small-cell lung cancers and cell lines and was associated with poor survival [16].

Furthermore, we investigated the roles of TNFR2 in pancreatic cancer. Firstly, we aim to explore the relationship between TNFR2 and clinicopathological data. We used the chi-square test to analyze clinicopathological data from pancreatic cancer patients and found that TNFR2 expression was positively correlated with aggressive clinicopathologic characteristics, including tumor size, lymph node metastasis, and differentiation. The clinicopathological data revealed that TNFR2 plays a significant role in PDAC proliferation, metastasis, and aggressiveness. Survival analysis showed that patients in the high-expression group had poor overall survival, suggesting that TNFR2 can serve as a prognostic marker.

To characterize the role of TNFR2 in pancreatic cancer, we used CCK-8, wound-healing, and Transwell assays. Compared to the NC group, proliferation, and metastatic capacity were suppressed in the KD group. These results suggest that TNFR2 enhances PDAC proliferation, migration, and invasion. Similar findings have been verified in other tumor studies. Mizoguchi et al. found that TNFR2 promoted the abnormal proliferation and malignant degeneration of inflammatory intestinal epithelial cells and contributed to the occurrence and development of colon cancer in a mouse model [17]. TNFR2 was essential in tumor angiogenesis and survival in highly vascularized murine lung tumor xenografts [18]. TNFR2 truly performs a vital role in the occurrence and development of tumors.

**Figure 5:**
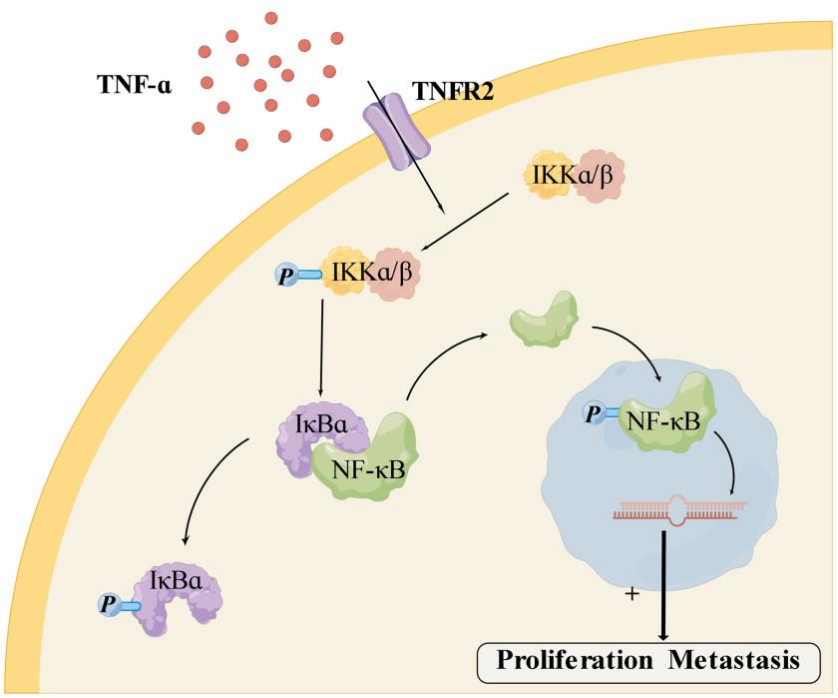
**Mechanism of TNFR2 and the NF-κB pathway** (generated using Figdraw http://www.figdraw.com).

We next investigated the underlying molecular mechanism of TNFR2. In the mid-19th century, Rudolf Virchow et al. found that inflammatory cells were abundant in tumor tissue, and cancer may develop from chronic inflammation [19]. Inflammation is a fundamental cancer-promoting factor [20, 21]. Chronic and persistent inflammation increases malignancy risk and acceleration of malignant progression in most cancers [22]. A recent study found that chronic inflammation enhanced chemotherapy resistance [23, 24]. NF-κB pathway is associated with chemotherapy, radiotherapy resistance, and tumor recurrence in various cancers, particularly in hormone-dependent breast cancer [25]. Other studies found that activation of the NF-κB pathway accelerated the transformation of chronic inflammation into tumors [26] and promoted tumor proliferation, migration, and invasion [27, 28]. Subsequently, we found that TNFR2 and the NF-κB pathway were overexpressed in PDAC cell lines. We silenced the TNFR2 gene using siRNA transient transfection. PDAC cells of NC and KD groups were cultured in a complete medium containing TNF-α after siRNA transient transfection, and western bolting showed that p-IKKα/β, NF-κB, p-NF-κB, p-IκBα protein decreased following TNFR2 silencing. TNF-α binds to TNFR2 on cell membranes, then activates downstream proteins of the NF-κB signaling pathway [12]. As shown in Figure 5, IKKα/β is phosphorylated to p-IKKα/β, which promotes the phosphorylation of IκBα to p-IκBα. Accompanied by the phosphorylation of IκBα, NF-κB detaches from IκBα and translocates into the nucleus. NF-κB transforms to p-NF-κB in the nucleus and contributes to PDAC proliferation and metastasis [29].

Our study demonstrated that TNFR2 overexpressed in pancreatic cancer. TNFR2 is highly expressed in human PDAC tissues and is stimulated by elevated TNF-α in the TME. TNFR2 significantly facilitates the proliferation and metastasis ability of PDAC cells via the NF-κB signaling pathway, which may contribute to the progression and metastasis of PDAC. TNFR2 may exacerbate inflammatory cell infiltration and catalyze chronic inflammation conversion to pancreas tumors. Currently, pancreatic cancer treatment is facing the dilemma of chemotherapy resistance. TNFR2 may be participating in chemotherapy drug resistance of pancreatic cancer. Treatment targeting TNFR2 may alleviate inflammatory cell infiltration in the tumor immune microenvironment, inhibit tumor cell proliferation and metastasis, and reverse drug resistance. Ultimately, our study provides a theoretical basis for the clinical application of therapy targeted toward TNFR2.

There are several limitations to this study. Firstly, the mechanisms by which TNFR2 acts were not verified *in vivo*. Secondly, the small sample size and low follow-up numbers of patients might limit the study’s statistical power. In a future study, we will expand the sample size and improve the follow-up. In addition to the NF-κB pathway, TNFR2 activates other transcription factors and signaling pathways, including PI3K, STAT3, and MAPK [30, 31]. Hamilton et al. determined that TNFα-induced TNFR2 promoted colon cancer progression via STAT3 [32]. Zhao et al. suggested that TNFR2 significantly promotes colon cancer via the PI3K/AKT signaling pathway [33]. These findings suggest that the underlying signaling mechanisms of TNFR2 in PDAC require study. Recent studies have designed and synthesized several antagonists of TNFR2. Preclinical studies have shown that mouse monoclonal anti-TNFR2 antibody achieves better efficacy when used as an antitumor immune agent in combination with immunotherapy than monotherapy [34].

A phase 1a/1b study (NCT05238883) of HFB200301 (TNFR2 agonist antibody) is recruiting. This study tested the safety, tolerability, and efficacy of HFB200301 as a single agent and in combination with tislelizumab in patients with advanced cancers. In a phase I trial (NCT05781386), the safety, efficacy, and pharmacokinetic/pharmacodynamic characteristics of SIM1811-03 (TNFR2 monoclonal antibody) are evaluated in subjects with advanced tumors. The drug targeting TNFR2 is currently in the recruitment phase of clinical trials. Limited by the immunosuppressive microenvironment, immunotherapy has not achieved the desired efficacy in pancreatic cancer. Targeted TNFR2 therapy combined with immunotherapy may be a potentially effective therapeutic option.

## CONCLUSION

In summary, our study determined that TNFR2 is overexpressed in PDAC. High expression of TNFR2 was associated with aggressive clinicopathologic characteristics and predicted poor outcomes in PDAC patients. TNFR2 participates in the progression and metastasis of PDAC cells via the NF-κB signaling pathway. Our study provides a theoretical basis for the clinical application of therapy targeted toward TNFR2.
